# Modeling Human Visual Search in Natural Scenes: A Combined Bayesian Searcher and Saliency Map Approach

**DOI:** 10.3389/fnsys.2022.882315

**Published:** 2022-05-27

**Authors:** Gaston Bujia, Melanie Sclar, Sebastian Vita, Guillermo Solovey, Juan Esteban Kamienkowski

**Affiliations:** ^1^Laboratorio de Inteligencia Artificial Aplicada, Instituto de Ciencias de la Computación, Universidad de Buenos Aires – CONICET, Ciudad Autónoma de Buenos Aires, Argentina; ^2^Instituto de Cálculo, Universidad de Buenos Aires – CONICET, Ciudad Autónoma de Buenos Aires, Argentina; ^3^Maestría de Explotación de Datos y Descubrimiento del Conocimiento, Facultad de Ciencias Exactas y Naturales, Universidad de Buenos Aires, Ciudad Autónoma de Buenos Aires, Argentina

**Keywords:** visual search, eye movements, ideal observer, saliency maps, human behavior

## Abstract

Finding objects is essential for almost any daily-life visual task. Saliency models have been useful to predict fixation locations in natural images during a free-exploring task. However, it is still challenging to predict the sequence of fixations during visual search. Bayesian observer models are particularly suited for this task because they represent visual search as an active sampling process. Nevertheless, how they adapt to natural images remains largely unexplored. Here, we propose a unified Bayesian model for visual search guided by saliency maps as prior information. We validated our model with a visual search experiment in natural scenes. We showed that, although state-of-the-art saliency models performed well in predicting the first two fixations in a visual search task ( 90% of the performance achieved by humans), their performance degraded to chance afterward. Therefore, saliency maps alone could model bottom-up first impressions but they were not enough to explain scanpaths when top-down task information was critical. In contrast, our model led to human-like performance and scanpaths as revealed by: first, the agreement between targets found by the model and the humans on a trial-by-trial basis; and second, the scanpath similarity between the model and the humans, that makes the behavior of the model indistinguishable from that of humans. Altogether, the combination of deep neural networks based saliency models for image processing and a Bayesian framework for scanpath integration probes to be a powerful and flexible approach to model human behavior in natural scenarios.

## 1. Introduction

Visual search is a natural task that humans perform in everyday life, from looking for someone in a photograph to searching your favorite mug in the kitchen. The ability to find a target relies on our capability to gather visual information through a sequence of eye movements; that perform a discrete sampling of the scene. This sampling of information is not carried out on random points. Decades of research have shown that the gaze follows different strategies, and tries to minimize the number of steps needed to find the target (Yarbus, 1967; Tatler et al., 2010; Borji and Itti, 2014; Rolfs, 2015). Indeed, visual search is a quintessential example of the active sensing or sampling paradigm (Yang et al., 2016b; Gottlieb and Oudeyer, 2018).

Humans must perform a goal-directed exploration that follows different perceptual cues (Wolfe and Horowitz, 2017) to find their target or to decide their absence. These sequential decisions are a trade-off between exploration and exploitation actions. The exploration actions attempt to reduce uncertainty about the environment, and the exploitation actions are conducted by knowledge-based decisions (Najemnik and Geisler, 2005; Yang et al., 2016a; Schwartenbeck et al., 2019). In natural stimuli, the exploitation phase is highly relevant given the importance of the context for finding an object (Eckstein et al., 2006). For instance, when looking for a plate in a kitchen, regions where related objects are present, like a table or a kitchen cabinet, would be more likely to contain the target object. Predicting eye movements required to locate a target is a computationally-complex task because it must combine the bottom-up information capturing processes, top-down integration of information, and updating of expectations on each fixation.

A computationally easier task is to predict where people will fixate when freely exploring a scene. This problem is usually addressed with computational models that build *saliency maps* which identify regions of an image that draw our attention. The first saliency models were built based on computer vision strategies, that combined different filters over the image (Itti and Koch, 2001). Some of these filters were very general, such as a low-pass filter that gave the idea of the horizon (Itti et al., 1998; Torralba and Sinha, 2001), or more specific, such as the detection of high-level features like faces (Cerf et al., 2008). In recent years, deep neural networks (DNNs) have advanced the development of saliency maps. Many saliency models have incorporated pre-trained convolutional DNNs successfully to extract low-level and high-level features of the images (Cornia et al., 2016, 2018; Kummerer et al., 2017). These novel approaches were summarized on the MIT/Tuebingen's collaboration website (Kummerer et al., 2018). Saliency maps have been successful at predicting fixations locations. However, saliency alone cannot account for the sequential nature of gaze movements. For example, the predictive power of saliency maps decays after the first few fixations (Torralba et al., 2006). More recently, Boccignone et al. (2019) proposed a time-aware model for scanpath prediction, where they sampled fixations from a dynamic saliency map and found that this approach led to more human-like behavior.

In recent years, there have been attempts to predict visual search scanpaths in natural scenes. For instance, Zhang et al. (2018) used a greedy algorithm based on DNNs, that mimicked the behavior of the visual system by elaborating an attention map related to the search goal. Using a greedy algorithm implied forcing some known behaviors of human visual search, like inhibition-of-return, that arise naturally with longer-sighted objective functions. More recently, Gupta et al. (2021) incorporated a model for the fovea and showed how human behavior biases emerged from DNN models mainly due to the biases present in the training data. Based on a different approach, Yang et al. (2020) used inverse reinforcement learning to model a slightly different task, known as categorical visual search, where instead of showing a specific target, a category was mentioned.

Nowadays, there is growing interest in Bayesian models because of their success in decision-making or perceptual tasks (Knill and Pouget, 2004; Tenenbaum et al., 2006; O'Reilly et al., 2012; Meyniel et al., 2015; Rohe and Noppeney, 2015; Samad et al., 2015; Wiecki et al., 2015; Turgeon et al., 2016). In the case of visual search, Najemnik and Geisler (2005) proposed a model that predicted the location of the next fixation based on its prior knowledge, a visibility map, and the current state of a posterior probability that was updated after every fixation. In this model, inhibition-of-return, moderate saccade length, among other human characteristics of visual search arose naturally (Najemnik and Geisler, 2005). The results of this Ideal Bayesian Searcher (IBS) have had a wide impact, but the images they used in their experiments were all artificial. Recently, Hoppe and Rothkopf (2019) proposed a visual search model that incorporated planning. He used the uncertainty of the current observation to select the upcoming gaze locations to maximize the probability of detecting the location of the target after the sequence of two saccades. For Najemnik and Geisler (2005), their task was designed specifically for maximize the difference between models (i.e., finding the target in very few fixations on artificial stimuli). To extend these results to natural images, it is necessary to incorporate the information available in the scene.

Here, we aimed to extend the IBS to natural images. To do that, we proposed to use state-of-the-art saliency maps as priors, and continuous similarity maps between the target template and different regions of the image. The motivation for using the saliency as prior in a visual search task lies in the results of the flash-preview moving-window paradigm. This paradigm showed that even a glimpse of less than a few hundreds of milliseconds of a scene guided search as long as sufficient time was available subsequently to combine prior knowledge with the current visual input (Oliva and Torralba, 2006; Torralba et al., 2006; Castelhano and Henderson, 2007). These modifications incorporated the first gist of the image and the notion of distractors. Finally, to validate our model we collected eye movement data from a visual search experiment in natural images and compared the overall performance between humans and the models, as most previous work has done, but also the entire scanpaths produced by the model with the ones recorded by human observers.

## 2. Materials and Methods

### 2.1. Participants

Fifty-seven subjects (34 male, 23 female; age 25.1 ± 5.9 years old) participated in the Visual Search task. All were students or teachers from the University of Buenos Aires. All subjects were naïve to the experiment's objectives, had normal or corrected-to-normal vision, and provided written informed consent according to the recommendations of the declaration of Helsinki to participate in the study. All the experiments described here were reviewed and approved by the ethics committee: “Comite de Ética del Centro de Educación Médica e Investigaciones Clínicas ‘Norberto Quirno' (CEMIC)” [qualified by the Department of Health and Human Services (HHS, USA): IRb00001745—IORG 0001315; Protocol number 435].

### 2.2. Paradigm and Procedure

Participants had to search for an object in a crowded indoor scene. Each trial started when the target was presented in the center of the screen, which subtended 144·144 pixels of visual angle (**Figure 2A**). After 3 s, the target was replaced by a fixation dot at a pseudo-random position at least 300 pixels away from the actual target position in the image (**Figure 2A**). This was done to avoid starting the search too close to the target. The initial position was the same for a given image and all participants. The search image appeared after the participant fixated the dot. Thus, all observers initiated the search in the same place for each image (**Figure 2A**). The image was presented at a 768 × 1, 024 resolution (subtending 28.3·28.8 degrees of visual angle; **Figure 2A**).

Saccades and fixations were parsed online. The search period finished when the participant fixated the target or after *N* saccades, with an extra 200 ms to allow observers to process information of this last fixation (Kotowicz et al., 2010). The maximum number of saccades allowed (*N*) were 2 (13.4% of the trials), 4 (14.9%), 8 (29.9%), or 12 (41.8%). These values were randomized for each participant, which was independent of the image. The experiment was programmed using PsychToolbox and EyeLink libraries in MATLAB (Brainard, 1997; Kleiner et al., 2007).

After each trial, participants were forced to guess the position of the target, even if they had already found it. They were instructed to cover the target position with a Gaussian blur, first by clicking on the center and then by choosing its radius. This was done by showing a screen with only the frame of the image and a mouse pointer—a small black dot—to select the desired center of the blur (**Figure 2A**). After the participant selected a position with the mouse, a Gaussian blur centered at that position was shown, and the participants were required to indicate the uncertainty of their decision by increasing or decreasing the size of the blur using the keyboard. The position and the uncertainty reports were not analyzed in the present study.

A training block of five trials was performed at the beginning of each session with the experimenter present in the room. After the training block, the experiment started and the experimenter moved to a contiguous room. The images were shown in random order. Each participant observed the 134 images in three blocks. Before each block, a 9-point calibration was performed, and the participants were encouraged to get a small break to allow them to rest between blocks. Moreover, each trial started with the built-in drift correction procedure from the EyeLink Toolbox, in which the participant had to fixate in a central dot and hit the spacebar to continue. If the gaze was detected far from the dot, a beep signaled the necessity of a re-calibration. The experiment was programmed using PsychToolbox and EyeLink libraries in MATLAB (Brainard, 1997; Kleiner et al., 2007).

### 2.3. Stimuli

The images corresponded to 134 indoor pictures from Wikimedia Commons, indoor design blogs, and LabelMe database (Russell et al., 2008), which have several objects and no human figures or text. The selection criterion was that scenes had several objects, and no human figures or text was presented. Moreover, the images were in black and white to make the task take more saccades, because color is a very strong bottom-up cue. Also, a pilot experiment with five participants was performed to select images that usually required several fixations to find the target. The size of the original images were all of at least 1, 024 × 768 pixels (subtending 28.3 × 28.8 degrees of visual angle), and all were cropped and/or scaled to 1, 024 × 768 pixels. For each image, a single target was selected manually among the objects of the size of at most 72 × 72 pixels—because we were not evaluating the accuracy of memory retrieval—. For all targets, we considered a surrounding region of 72 × 72 pixels. Finally, we checked that there were no consistent spatial biases across the images (Supplementary Figure 2).

### 2.4. Data Acquisition

Participants were seated in a dark room, 55*cm* away from a 19-inch Samsung SyncMaster 997 MB monitor (refresh rate = 60 Hz), with a resolution of 1, 280 × 960. A chin and forehead rest was used to stabilize the head. Eye movements were acquired with an Eye Link 1000 (SR Research, Ontario, Canada) monocular at 1,000 Hz.

### 2.5. Data Preprocessing

The saccade detection was performed online with the native EyeLink algorithm with the default parameters for cognitive tasks. Fixations were collapsed into a grid with cells of 32 × 32 pixels, which resulted in a grid size of 32 × 24 cells. We explored the size of the grid in terms of model performance. Consecutive fixations within a cell were collapsed into one fixation to be consistent with the model behavior. Also, fixations outside the image region were displaced to the closest cell. Because we considered fixations, blinks periods were excluded.

The trial was considered correct (i.e., target found) if the participant fixated into the target region (72 × 72 pixels). Only correct trials were analyzed in terms of eye movements.

Participants completed (71 ± 17) trials (or images), which corresponds to (30 ± 13) trials (or participants) per image. This results in a total of 4,054 out of 7,455 (54.3%) successful trials considered in the scanpath similarity analysis. The unsuccessful trials corresponded mainly to the low saccade threshold trials as can be observed in **Figure 4A** (which were randomly distributed along the images).

### 2.6. Models

#### 2.6.1. Saliency Maps

In the last few years, several saliency models appeared in the literature and made their code available. Many of those were summarized nicely and compared in the https://saliency.tuebingen.ai/ repository (Judd et al., 2012; Bylinskii et al., 2018; Kummerer et al., 2018). With the purpose of understanding which features guided the search, we chose and compared five different state-of-the-art saliency maps for our task: DeepGazeII (Kummerer et al., 2017), MLNet (Cornia et al., 2016), SAM-VGG and SAM-ResNet (Cornia et al., 2018), and ICF (Intensity Contrast Feature) (Kummerer et al., 2017).

All the saliency models considered (except for ICF) were based on neural network architectures that used different convolutional networks (CNN) pretrained on object recognition tasks. These CNNs played the role of calculating a fixed feature space representation (feature extractor) for the image that was then be fed to a predictor function (in the models we considered, also a neural network). DeepGaze II used a VGG-19 (Simonyan and Zisserman, 2014) as feature extractor, and the predictor was a simpler four-layer CNN (Kummerer et al., 2017). The MLNet model used a modified VGG-16 (Simonyan and Zisserman, 2014) that returned several feature maps, and a simpler CNN is used as a predictor that incorporated a learnable center prior (Cornia et al., 2016). Finally, SAM used both VGG-16 and ResNet50 (He et al., 2016) as two different feature extractors, and the predictor was a neural network with attentive and convolutional mechanisms (Cornia et al., 2018). ICF had a similar architecture to DeepGaze II, but it used Gaussian filters instead of a neural network. This way, ICF extracted purely low-level image information (intensity and intensity contrast). We also included a saliency model with just the center bias that was modeled by a 2D Gaussian distribution.

As the control model, we built a human-based saliency map using the accumulated fixation position of all observers for a given image, which was smoothed with a Gaussian kernel (st. dev. = 25 pxs). Given that observers were forced to begin each trial in the same position, we did not use the first fixations but the third. This way we captured the regions that attracted human attention.

#### 2.6.2. Bayesian Searcher

To predict human visual search scanpaths, Najemnik and Geisler (2005) proposed the Ideal Bayesian Searcher (IBS). The IBS considered each possible next fixation sequentially and picked the one that maximized the probability of identifying the location of the target correctly after the fixation. The decision of the optimal fixation location at step *T* + 1, *k*_*opt*_(*T* + 1), was computed as (Equation 1):


(1) $$\begin{array}{l} k_{opt}\left(T+1\right)=\underset{k\left(T+1\right)}{\text{arg max }}\left\{p\left(C|k\left(T+1\right)\right)\right\} \end{array}$$

This rule was rewritten by conditioning on the location *i* as:


(2) $$\begin{array}{l} k_{opt}\left(T+1\right)=\underset{k\left(T+1\right)}{\text{arg max }}\left\{\overset{n}{\underset{i=1}{\sum }}p_{i}\left(T\right)p\left(C|i,k\left(T+1\right)\right)\right\} \end{array}$$

where *p*_*i*_(*T*) was the posterior probability that the target was at the *i*-th location within the grid after *T* fixations and *p*[*C*|*i, k*(*T* + 1)] was the probability of being correct given that the true target location was *i*, and the location of the next fixation was *k*(*T* + 1). To compute Equation (2), Najemnik and Geisler (2005) derived a formulation that allowed us to estimate *p*[*C*|*i, k*(*T* + 1)] (see Supplementary Material). Then, we computed for each possible next location what we called the *detectability map*: $\sum_{i=1}^{n}p_{i}\left(T\right)p\left[C|i,k\left(T+1\right)\right]$ (as an example see Supplementary Figure 1). The posteriors, *p*_*i*_(*T*), involved the prior, the visibility map [$d_{ik\left(t\right)}^{\prime }$] and a notion of the target location [*W*_*ik*(*t*)_]:


(3) $$\begin{array}{l} p_{i}\left(T\right)=\frac{prior\left(i\right)\;\cdot \;\overset{T}{\underset{t=1}{\prod }}exp\;(d_{ik\left(t\right)}^{\prime 2}W_{ik\left(t\right)})}{\overset{n}{\underset{j=1}{\sum }}prior\left(j\right)\;\cdot \;\overset{T}{\underset{t=1}{\prod }}exp\;(d_{jk\left(t\right)}^{\prime 2}W_{jk\left(t\right)})} \end{array}$$

The template response, *W*_*ik*(*t*)_, quantified the evidence gathered from a given position *i* about the target image when the observer is fixated at position *k*(*t*) (*t* was any previous fixation). It was defined as $W_{ik\left(t\right)}\sim N\left(\mu_{ik\left(t\right)},\sigma_{ik\left(t\right)}^{2}\right)$ where:


(4) $$\begin{array}{l} \mu_{ik\left(t\right)}=1_{\left(i\;=\;target\;location\right)}-0.5\;,\;\sigma_{ik\left(t\right)}=\frac{1}{d_{ik\left(t\right)}^{\prime }} \end{array}$$

Abusing notation, in Equation (3) *W*_*ik*(*t*)_ referred to a value drawn from this distribution. IBS has only been tested in artificial images, where subjects needed to find a Gabor patch among 1/*f* noise in one out of 25 possible locations. This work is, to our knowledge, the first one to test this approach in natural scenes. Below, we discuss the modifications needed to apply IBS to eye movements in natural images.

#### 2.6.3. Modifications to the IBS to Handle Natural Scenes

Because it would be both computationally intractable to compute the probability of fixating in every pixel of a 1, 024 × 768 image, and ineffective to do so—as useful information spans over regions larger than a pixel—, we restricted the possible fixation locations to be analyzed to the center points of a grid of δ × δ pixels each. We collapsed the eye movements to these points accordingly: consecutive fixations within a cell were merged into one fixation to be consistent with the model behavior.

The original IBS model had a uniform prior distribution. Because we were trying to model fixation locations in a natural scene, we introduced a saliency model as the prior. The *prior*(*i*) was the average of the saliency in the *i*-th grid cell.

Importantly, the presence of the target in a certain position in natural images was not as straightforward as in artificial stimuli, where all the incorrect locations were equally dissimilar. In natural images there are often distractors (i.e., positions in the image that are visually similar to the target, especially if seen with low visibility). Therefore, we proposed a redefined template response ${\tilde{W}}_{ik\left(t\right)}$ as Equation (5), and ${\tilde{\mu }}_{ik\left(t\right)}\in \left[-1,1\right]$ was defined as Equation (6).


(5) $$\begin{array}{l} {\tilde{W}}_{ik\left(t\right)}\sim N\left({\tilde{\mu }}_{ik\left(t\right)},{\tilde{\sigma }}_{ik\left(t\right)}^{2}\right) \end{array}$$

(6) $$\begin{array}{l} {\tilde{\mu }}_{ik\left(t\right)}=\mu_{ik\left(t\right)}\cdot \left(d_{ik\left(t\right)}^{\prime }+\frac{1}{2}\right)+\phi_{i}\cdot \left(\frac{3}{2}-d_{ik\left(t\right)}^{\prime }\right) \end{array}$$

where ϕ_*i*_ quantified how similar is each location *i* to the target, i.e., the *similarity map*. Here, we proposed two alternatives: (1) to use the cross-correlation between the target template and the location that we called the *correlation* IBS or cIBS model, or (2) to use the *structural-similarity* (Wang et al., 2004), the sIBS model.

Also, we included a model of the visibility map. The parameters of the 2D Gaussian model were chosen *a priori* and estimated from values reported in Najemnik and Geisler (2005) and Bradley et al. (2014), and they were the same for every participant. The bivariate Gaussian N(μ,Σ) was centered on each fixation point (**μ** is the 2D-coordinate in pixels), and its covariance was $\Sigma =\left(\begin{array}{cc} 2600 & 0\\ 0 & 4000 \end{array}\right){\text{pxs}}^{2}$. Moreover, we modified σ_*ik*(*t*)_ to keep the variance depending on the visibility, but we incorporated two parameters (Equation 7):


(7) $$\begin{array}{l} {\tilde{\sigma }}_{ik\left(t\right)}=\frac{1}{a\;\cdot \;d_{ik\left(t\right)}^{\prime }+b} \end{array}$$

The parameters *a* and *b* jointly modulated the inverse of the visibility and prevented 1/*d*′ from diverging. These parameters were not included in the original model probably because *d*′ was estimated empirically (from thousands of trials and independently for each subject) and the *d*′ was never exactly equal to zero. Bradley et al. (2014) simplified the task by fitting a visibility map built from a first-principle model that proposed an analytic function with several parameters, which should still be fitted for each participant. Here, we further simplified it by using a two-dimensional Gaussian with the same parameters for every participant, which avoided a potential leak of information about the viewing patterns to the model. The parameters were taken *a priori* (estimated from parameters in Najemnik and Geisler, 2005; Bradley et al., 2014). We chose the parameters of the model using a classical grid search procedure in a previous experiment with a smaller dataset and the same best parameters (δ = 32, *a* = 3, and *b* = 4) were used for all the models.

### 2.7. Metrics

#### 2.7.1. Mean Agreement

To compare the performance of models against humans, we calculated the mean proportion of trials where both the participant and the model had the same behavior [i.e., both found (or not) the target under the same saccades thresholds]. We call this measure Mean Agreement, and it quantifies the compromise between our model and the participants in their performance. The higher the value of MA was, the better.

#### 2.7.2. MultiMatch

MultiMatch (Dewhurst et al., 2012) is a multi-dimensional similarity measure between scanpaths that is composed of five different similarity metrics: shape(vector), direction, length, position, and duration. Each of them compares a specific characteristic of the scanpaths producing a values between 0 (worst similarity) and 1 (best similarity, identical characteristic). The *shape* metric is the difference between aligned saccade pairs and the *position* is the Euclidean distance between aligned fixations. The *length* similarity measures the difference in length of each saccade and the *direction* compares the angular distance between the saccades' directions. Each of these metrics is normalized and transformed into a similarity when needed. We use the original implementation in MATLAB with its default parameters.

The duration similarity was not taken into account because our model was not designed to predict it. For MM, the duration of the fixations is used in only for two purposes: simplifying the scanpaths (group fixations together) and, for calculating the duration similarity (Jarodzka et al., 2010; Dewhurst et al., 2012). In our case, we first simplify our scanpaths, and then we map them to a grid previously to calculate MM. This way, not considering the fixation's duration does not affect the other four MultiMatch metrics. For the comparison, each metric could be considered separately or as a composite variable (AvMM), in which case, we do not considered the fixation duration.

All MM values presented in Table 1 represent the mean value of the pairwise MultiMatch similarity between the scanpaths. When comparing humans' results, the mean was taken over all subjects' scanpaths when the target was found: *within-humans* MultiMatch (whMM). For each model, we took the mean over all pairwise MultiMatch scores between subjects and models where the target was found: *human-model* MultiMatch (hmMM).

**Table 1:** Models results.

**Searcher**	**Prior**	**MA (SD)**	**Corr**	**Slope**	**AvMM**	**MMvec**	**MMdir**	**MMlen**	**MMpos**
Humans		0.65 ± 0.08	–	–	0.83	0.88	0.73	0.89	0.83
sIBS	DGII	**0.65 ± 0.09**	**0.48**	1.00	**0.83**	**0.88**	**0.71**	**0.83**	0.90
sIBS	Center	0.60 ± 0.09	0.34	1.00	0.82	**0.88**	0.65	0.82	**0.91**
sIBS	Flat	0.62 ± 0.09	0.06*	1.02	0.81	0.86	**0.71**	0.80	0.87
sIBS	Noisy	0.63 ± 0.07	−0.14*	1.03	0.78	0.84	0.65	0.77	0.87
cIBS	DGII	0.65 ± 0.08	0.40	1.00	**0.83**	**0.88**	0.70	**0.83**	0.90
cIBS	Center	0.59 ± 0.90	0.28	1.00	0.82	**0.88**	0.66	**0.83**	**0.91**
cIBS	Flat	0.60 ± 0.09	0.27	1.04	0.80	0.85	0.70	0.80	0.85
cIBS	Noisy	0.59 ± 0.09	0.18*	1.04	0.78	0.85	0.66	0.76	0.87
IBS	DGII	0.64 ± 0.10	0.18	0.99	**0.83**	**0.88**	**0.71**	**0.83**	0.90
IBS	Center	0.62 ± 0.08	0.20*	1.01	0.82	0.88	0.65	0.82	**0.91**
IBS	Flat	0.63 ± 0.11	0.25	1.02	0.81	0.86	0.71	0.81	0.87
IBS	Noisy	0.63 ± 0.09	0.03*	1.04	0.78	0.84	0.65	0.77	0.87
greedy	DGII	0.62 ± 0.08	0.09*	0.99	**0.83**	**0.88**	**0.71**	**0.83**	0.90
SS	DGII	0.55 ± 0.09	0.38	1.04	0.78	0.84	0.59	0.78	0.89

Mean Agreement measures the coincidence of humans and model's correct target detection. slope corresponds to the slope of a linear regression y ~ x model between the similarity of human scanpaths (whMM in **Figures 4C**, **5C**) and the similarity of model's scanpaths (hmMM). corr is the Pearson's correlation coefficient between humans and model's scanpath, which are whMM and hmMM, respectively. Only correct trials were considered and averaged for each image (N = 134). Also, average MultiMatch dimensions are reported: vector (MMvec), direction (MMdir), length (MMlen), position (MMpos), and also global average MultiMatch (AvMM). All correlation values were significantly different from 0 (pval < 0.05), except those marked as (*). All slopes were significantly different from 0 (pval < 10^−8^). The bold numbers indicate the best performing model in each metric.

#### 2.7.3. Correlation and Linear Model

To summarize and to visualize the comparison of scanpaths, we calculated the Pearson correlation between the whMM pairwise values and the hmMM values. We also reported the slope of a linear regression whMM ~ hmMM without intercept.

## 3. Results

### 3.1. Searcher Modeling Approach

**Figure 1:**
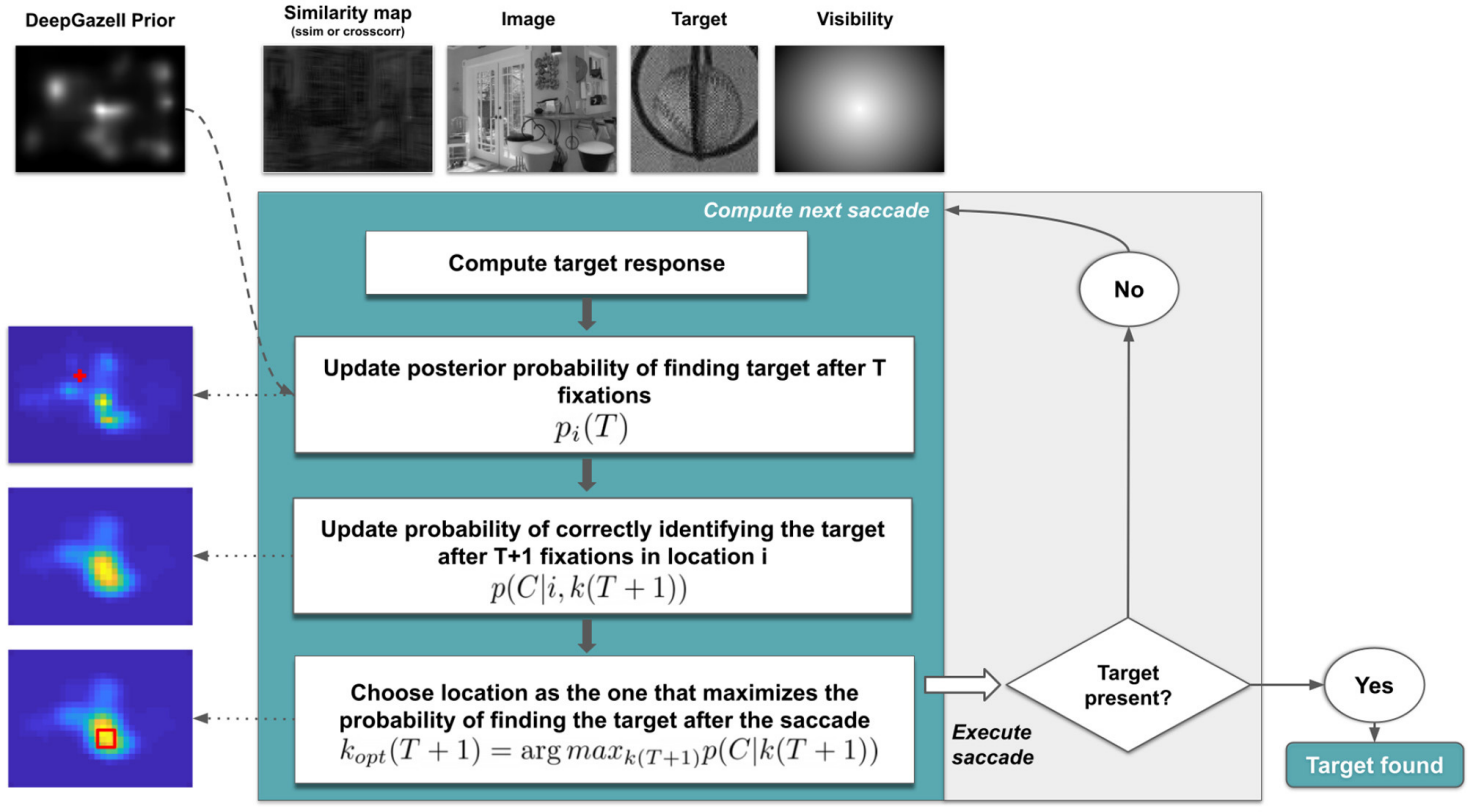
Bayesian model scheme. Simplified outline of the main framework. To compute each saccade (cyan box), in the first instance, the target response (first white box) is precalculated for each possible location (*W*) using the selected similarity: presence/absence of the target **IBS**, cross-correlation **cIBS**, or structural similarity **sIBS**. Then, the computation of next saccade has two main steps: 1. estimate the probability of finding the target on current fixation after *T* saccades (second white box) and 2. estimate the probability of correctly finding the target on next fixation *T* + 1 (third white box) given the current knowledge of the image and the current fixation. Those estimations as 2D maps with a red cross that represents the current fixation (left panels). Next, the model picks the location that maximizes the probability of finding correctly the target (fourth white box). Finally, the saccade is executed and if the target is present on the new fixation location the search stops but if it is not present, the next saccade is calculated. More details can be found on Supplementary Material.

According to the IBS proposed by Najemnik and Geisler (2005), an observer will make a saccade to a location that maximizes the probability of detecting the target at each location, taking into account a visibility map and previous fixations (further details in Section 2.6). Our approach expanded this model to a visual search task in natural domains (Figure 1). It involved two main aspects: (1) A prior distribution (*saliency map*) estimation step as a first, glimpse-like, information extraction, and then, (2) successive search steps were performed to build the full scanpath. Briefly, in each step, the model selected the next fixation over a grid and decided if the target was positively there using structural similarity (Wang et al., 2004) between the target and the location (**sIBS**) or using cross-correlation (**cIBS**).

### 3.2. Paradigm and Human Behavior Results

**Figure 2:**
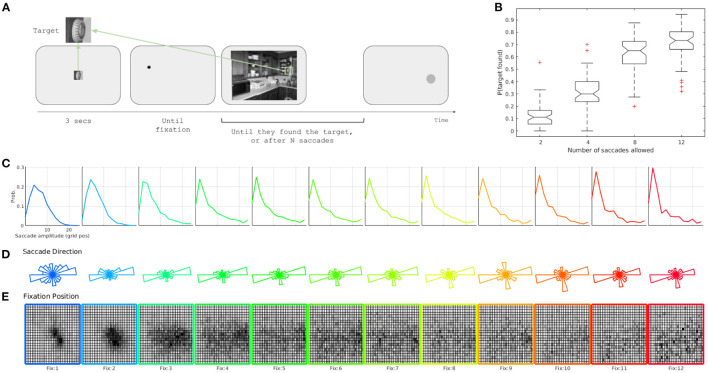
Paradigm and general behavior. **(A)** Experimental schema. **(B)** Proportion of targets found as a function of the number of saccades allowed. Distributions of **(C)** saccade length, **(D)** saccade direction (measured in degrees from the positive horizontal axis), and **(E)** location of fixations for different fixation ranks.

To test our model, we ran a visual search experiment in which the participants had to search for a cropped target object within a natural indoor scene. The trial stopped when the participant found the target or after *N* saccades (*N* = 2, 4, 8, 12). As expected, the proportion of targets found increased as a function of the saccades allowed (Figure 2A), which reached a plateau from 8 to 12 saccades (Figure 2A and data from a preliminary experiment with up to 64 saccades not shown).

Overall, eye movements that we have recorded behave as expected. First, the amplitude decreased with fixation rank, which presented the so-called coarse-to-fine effect (Figure 2B), and the saccades tended to be more horizontal than vertical (Figure 2C). Finally, the initial spatial distribution of fixations had a central bias, and then they extended first over the horizon until it covered the entire image, because the targets were distributed uniformly throughout the scene (Figure 2D). This effect could be due partially to the organization of the task (i.e., the central drift correction and presentation of the target), the setup (i.e., the central position of the monitor with respect of the eyes/head), and the images (i.e., the photographer typically centered the image); it also could be due to processing benefits because it was the optimal position to acquire low-level information of the entire scene or to start the exploration.

### 3.3. Exploring Saliency Maps

**Figure 3:**
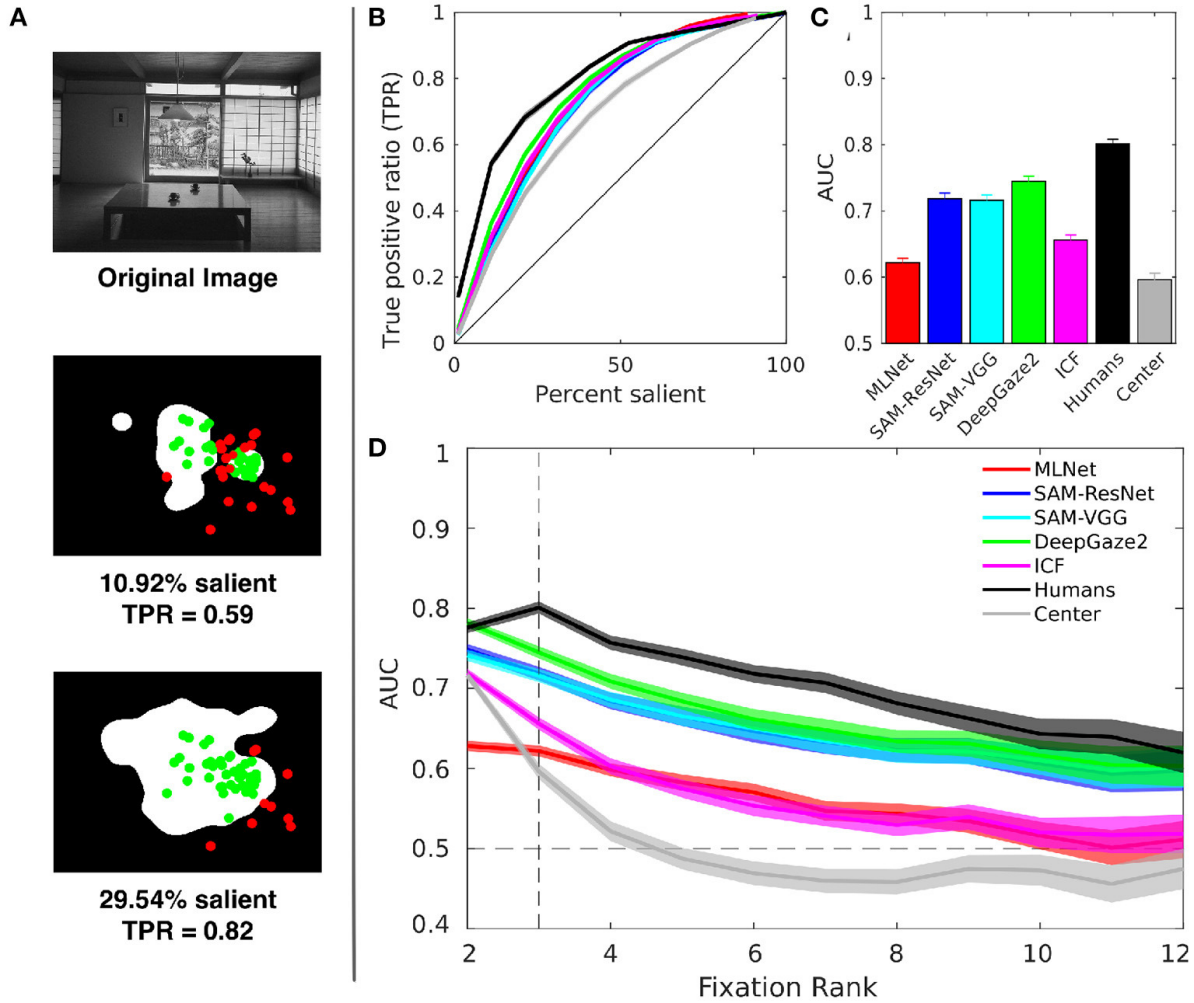
Saliency maps. We compare the results of five state-of-the-art models: DeepGazeII, ICF (Kummerer et al., 2017), SAM(ResNet-50 and VGG-16 versions) (Cornia et al., 2018), and MLNet (Cornia et al., 2016), and two controls: a center bias (2D centered Gaussian) and an empirical third-fixation subjects saliency map (humans). **(A)** Example of how to estimate the TPR for the ROC curve, **(B)** ROC curves, **(C)** AUC-Borji values for the third fixation, and **(D)** AUC for each model as a function of the current Fixation Rank. Color mapping for models is consistent for **(B–D)**.

As a first approach, we evaluated how saliency models performed by themselves to predict fixations along the search. This evaluation served as a baseline performance for our model. We considered each saliency map **S** as a binary classifier on every pixel and used Receiver Operator Curves (ROC) and Area Under the Curve (AUC) to measure their performance. There was not a unique way of defining the false positive rate (**fpr**). In dealing with this problem, previous work on this task used many different definitions of ROC and its corresponding AUC (Borji et al., 2013; Riche et al., 2013; Bylinskii et al., 2018; Kummerer et al., 2018). Briefly, to build our ROC we considered the true positive rate (**tpr**) as the proportion of saliency map values above each threshold at fixation locations and the **fpr** as the proportion of saliency map values above each threshold at non-fixated pixels (Figure 3A).

As expected, the saliency map built from the distribution of third fixations performed by humans (human-based saliency map) was superior to all other saliency maps, and the center bias map was clearly worse than the rest of them (Figure 3B). This was consistent with the idea that the first steps in visual search were mostly guided by image saliency. The rest of the models had similar performances on AUC, although DeepGazeII performed slightly better than the others (Figures 3B,C).

All models reached a maximum in AUC values at the second fixation except the human-based model that peaked at the third fixation, as expected (Figure 3D). Interestingly, the center bias began at a similar level as the other saliency maps, but decayed more rapidly; it reached 0.5 in the fourth fixation. Thus, other saliency maps must have captured some other relevant visual information. Nevertheless, the AUC values from all saliency maps decayed smoothly (Figure 3D), which suggested that the gist the observers were able to collect in the first fixations was modified largely by the search. Top-down mechanisms might have taken control and played a major role in the guidance of eye movements as the number of fixations increased (Itti and Koch, 2000). Moreover, we observed that the fixations spread away from the center as the trial progress (Figure 2E), therefore it seems not to be just the center bias attracting fixations that could explained the AUC decay as in other studies (Tatler et al., 2005) which could be an alternative explanation. The DeepGazeII model performed better over all fixation ranks, and it became indistinguishable from human performance in the second fixation (Figure 3D).

### 3.4. Evaluating Searcher Models on Human Data

**Figure 4:**
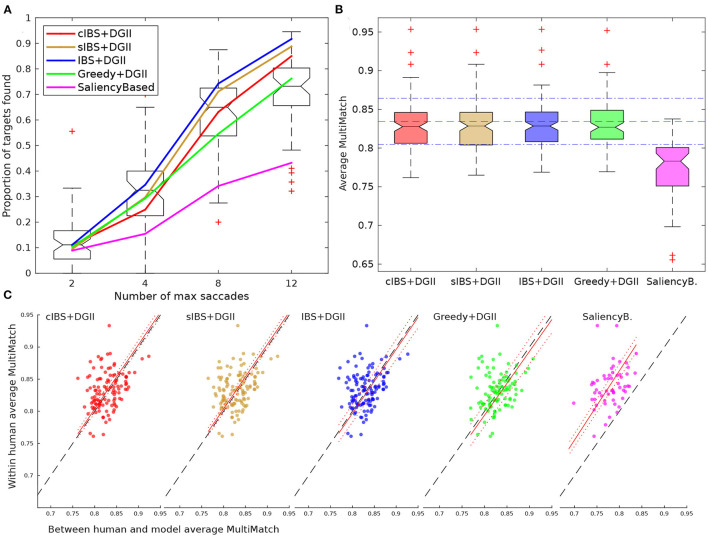
Searcher strategy. **(A)** Proportion of targets found for each threshold that we considered. Human behavior is presented as a boxplot. The performance achieved by the models using different search strategies and DeepGazeII (DGII) as a prior is presented as curves. **(B)** Distribution of average MultiMatch similarity scores (vector, direction, position and length) for each strategy (higher is better). Blue horizontal lines represent the mean and standard deviation scores for humans (0.83 ± 0.02). **(C)** Mean within-human MultiMatch scores against the mean between human and the model's MultiMatch scores. Linear regression with *intercept* = 0 was performed (red lines; see Table 1). Each dot represents an image of the dataset. Only trials where the target was found are considered in **(B,C)** for computing MultiMatch score.

**Figure 5:**
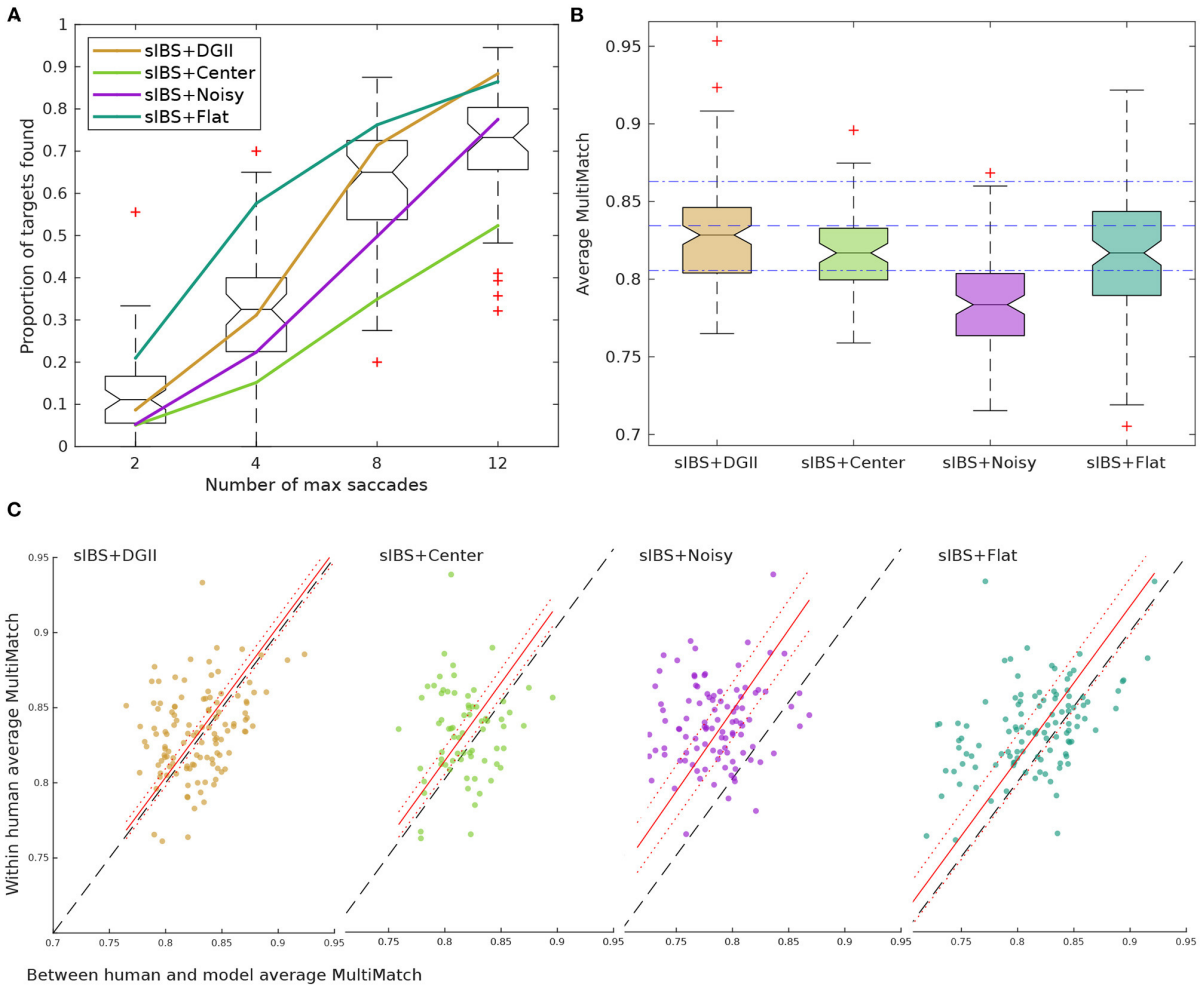
Saliency maps as priors. **(A)** Proportion of targets found for each threshold considered. Human behavior is presented as a boxplot. The performance achieved by the models using sIBS with different prior distributions. **(B)** Distribution of average MultiMatch similarity score (vector, direction, position, and length) for each strategy (higher is better). Blue horizontal lines represent the mean and standard deviation scores for humans (0.83 ± 0.02). **(C)** Mean within-human MultiMatch scores against the mean between human and models MultiMatch scores. Linear regression with *intercept* = 0 was performed (red lines; see Table 1). Each dot represents an image of the dataset. Only trials where the target was found are considered in **(B,C)** for computing MultiMatch score.

As expected, saliency maps alone were not able to follow the fixations after the first few. Therefore, we moved forward with the proposed IBS model. We first evaluated the updating of probabilities and the decision rule for the next fixation position of the two proposed variants of the model, the *sIBS* and *cIBS* models. For comparison, we used the previous *IBS* model, in which the *template response* accounted only for the presence or absence of the target and not for the similarity of the given region with the target (Figure 4). Also, we implemented two other control models: a *Greedy searcher* and a *Saliency-based searcher*. The *Greedy searcher* based its decision to maximize the probability of finding the target in the next fixation. It only considered the present posterior probabilities and the visibility map, and it did not take into account how the probability map was going to be updated after that. The *Saliency-based searcher* simply went through the most salient regions of the image, and it added an inhibition-of-return effect to each visited region. In these models, we used the DeepGazeII as a prior because it was the best performing saliency map of the previous section. We also evaluated the usage of different priors with the sIBS model; we compared this with the center bias alone, which had a centered two-dimensional Gaussian distribution, a uniform (flat) distribution, and a white noise distribution (Figure 5).

The overall performance in the task was calculated as the proportion of targets found for different saccade thresholds (Figure 4A), and the difference between models was measured as the proportion of trials where subject and model had the same performance [i.e., both found (or not) the target (*Mean Agreement*)]. When comparing different searchers with the same prior (DeepGazeII), sIBS had the best agreement (0.65 ± 0.09) with the humans' performance (Table 1). Nevertheless, the curves of the cIBS, IBS, and Greedy models were also very close to humans (Figure 4A), and each of them reached a similar performance on Mean Agreement, 0.65, 0.64, and 0.62, respectively (Table 1). The IBS model had the best performance curve, and it found the target in fewer saccades than all the other models, while the inclusion of the template matching variants (sIBS and cIBS) resulted in a more human-like performance. Only the control Saliency-based model had a markedly poorer agreement with the human performance (0.55 ± 0.09), which showed that template matching weighted by visibility was a plausible mechanism for searching potential targets in the scene.

Furthermore, we compared the entire scanpaths between subjects and models using the MultiMatch (MM) score (Dewhurst et al., 2012). As mentioned in 2.7.2, this metric measures the similarity between two scanpaths with five different scores that compared different characteristics of the scanpaths: *vector, direction, length, position*, and *duration*. The duration similarity was not included because we were not predicting duration of fixations. The distribution of the average MM between humans and models for each image (hmMM), except for the Saliency-based model showed a similar value (Figure 4B and Table 1). Moreover, these four models fell within the mean ± standard deviation of average MM within-humans for each image (whMM) (Figure 4B, blue lines). The variability in the MM scores was explained partially by the image, thus, we also compared the correlation between hmMM and whMM for the different images (Figure 4C), and obtained a larger correlation with the sIBS model (*r* = 0.48, Table 1).

Then, we explored the importance of the prior by comparing the best searcher model, sIBS, with different basic priors. The sIBS+DeepGazeII had better Mean Agreement with human behavior (0.65 ± 0.09; Figure 5A and Table 1). Interestingly, using DGII as prior was the only model that presented a step-like function characteristic of humans (Figure 5A). The center prior had the worst performance and also the lowest Mean Agreement with humans (0.60 ± 0.09). The flat prior achieved the best performance but the Mean Agreement with humans was lower than that achieved by DGII (0.62 ± 0.09). This suggested that the model using a flat prior behaved more like an optimal searcher, but this seemed not to be the case for humans (Zhou and Yu, 2021).

When comparing similarity of scanpaths, we observed that the models with DeepGazeII and Center priors were closer to humans' values than others, but the flat and noisy priors had lower scanpath similarities (average hmMM = 0.81 and 0.78, respectively; Figure 5B). The noisy prior hmMM distribution showed a drift in comparison with whMM, which resulted in a higher slope (1.03). This suggested that humans were, on average, more consistent than the model. Also, the flat and noisy prior achieved a lower correlation, and both the models with DeepGazeII and Center priors had the best correlation and slopes closer to 1 (Figure 5C and Table 1) which suggested that the initial center bias was a fair approximation of the human priors. Nonetheless, although scanpaths from both models were almost indistinguishable from humans, saliency added some information that made the model with DeepGazeII produce a behavior more similar to humans in both performance and MM.

### 3.5. Scanpath Examples

**Figure 6:**
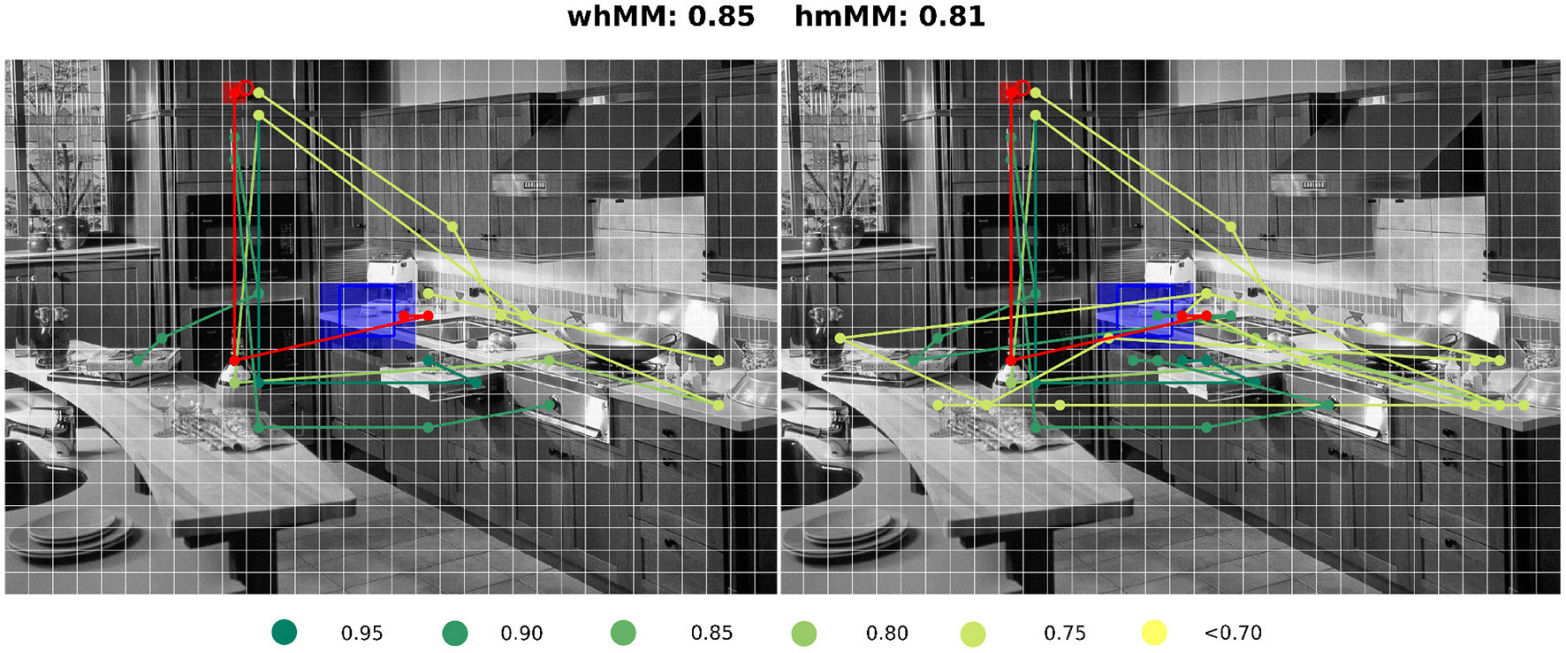
Comparison of scanpath predictions. The figure is meshed by a fixed grid of δ = 32px. Each curve represents a scanpath, in red the cIBS+DGII model's scanpath, and in green and yellow six scanpaths of participants colored according to their similarity to the model scanpath. The **(left)** panel shows the first four fixations of each scanpath, and the **(right)** panel shows the entire scanpaths. The search target is represented with the blue square and the approximated first fixation with the red square. Above the image, the whMM and hmMM for this trial are reported. Image taken from *Wikipedia Commons*.

When looking deeper into the images that had the lower similarity between model and participants, we observed that participants performed two or more different, but consistent, patterns. Because the present implementation of the model was deterministic, it chose only one of those patterns (Figure 6). In Figure 6, we illustrate this by showing some of the human scanpaths and the model (cIBS+DGII) scanpath for one image. In this case, the cup was the search target and there were two surfaces where, a priori, we were equally likely to find it. We selected six human scanpaths, the best three scanpaths in terms of hmMM, and from the worst six, we presented the three that fixated onto the other surface where they might find the target (not the one chosen by other subjects and the model. Note that dark green traces were scanpaths that were very similar to the model, and yellow traces are scanpaths that differed from the model. This behavior is not present in every image, and we aim to explore which aspects of the image or the previous scanpath trigger this behavior in future research.

## 4. Discussion

We introduced the sIBS and the cIBS models as expansions of the IBS model to face natural scenes. In summary, we used saliency maps as priors to model the information collected in the first glimpse that guided the first saccades, and we modified the computation of the *template response* to be able to, first, use a simpler model of visibility and, second, give graded responses to regions similar to the target to incorporate the notion of distractors. To evaluate the model, we created a dataset of 57 subjects that searched 134 images, where we compared both models to IBS and other strong baselines. We observed that saliency models performed well in predicting initial fixations, in particular the third fixation. Humans seemed to start from the initial forced fixation position, move to the center, and then to the most (bottom-up) salient location. After that, the performance of all the saliency models decayed to almost chance, as was expected from their conception. They mainly encoded bottom-up information of the image (Itti and Koch, 2000) and not the aim of the task, and they were not able to change or to update as it progressed. This was also consistent with previous results from Torralba et al. (2006) who implement a saliency model for a visual search task.

Because saliency models are good in predicting first bottom-up impressions, they are ideal candidates to be included as priors in the proposed Bayesian framework. The central bias performed well in the first two fixations, and it was included in all the saliency models (Cornia et al., 2016, 2018; Kummerer et al., 2017). The central bias by itself resulted in a good prior in terms of scanpath similarity, but not in the performance of finding the target. However, DeepGazeII had the better compromise of both measures. This suggested that bottom-up cues provided by saliency maps were relevant to the search. The Ideal Bayesian Observer, originally proposed by Najemnik and Geisler (2005), was an approximation to an optimal searcher that tries to minimize the number of steps taken to find the target. Nevertheless, the discussion on whether humans follow optimal or sub-optimal behavior when it comes to visual search is still present today (Zhou and Yu, 2021). It is likely that this behavior is strongly influenced by what kind of search is carried out, whether the stimuli are natural or artificial, or how the target is shown to subjects. The results obtained with sIBS, cIBS, and IBS using flat priors were more consistent with optimal behavior, but their results were not as similar to humans when compared with more sophisticated priors like DeepGazeII. The information encoded by saliency models had a substantial influence on the scanpaths that were predicted by these models and ensued a behavior more compatible with a sub-optimal strategy.

Regarding the update and decision rule, it was clear that the simple saliency-based searcher was not a good model of visual search. Humans use not only information about the scene but also information about the target and previous fixations. Thus, a rule of comparing peripheral information of the scene and the target should be implemented, along with a mechanism for combining that information. In the present study, those mechanisms were implemented in the Greedy, IBS, cIBS, and sIBS models, with the difference that the Greedy model maximized the probability of finding the target in the next fixation, and the rest maximized the probability of finding the target after the next fixation. The three Bayesian models using DGII as prior achieved the best metrics among all. No major differences were found between the two alternatives cIBS and sIBS, but the latter that had the best overall performance. The inclusion of different strategies for template matching was relevant to account for possible distractors present in the scene. Nevertheless, the Greedy achieved similar metrics to those achieved by the planning models.

Previous efforts that included contextual information aimed mainly to predict image regions that were likely to be fixated. For instance, they combined statically a spatial filter-based saliency map with previous knowledge of target object positions on the scene (Torralba et al., 2006). Some other research aimed to predict the sequence of fixations, but efforts on non-Bayesian modeling mainly used greedy algorithms (Rasouli and Tsotsos, 2014; Zhang et al., 2018). Here, we compared an example of a greedy algorithm with others with a more long-sighted objective function, which had the additional benefit of having some known behaviors of human visual search arise naturally. For example, Zhang et al. (2018) forced Inhibition-of-Return, but our model incorporated it implicitly. Briefly, this behavior on the location *i* was achieved in the proposed model because the visibility was maximum when the display location was the same as the one currently being observed (*i*), so it followed that *W*_*i,k*(*t*) = *i*_ = *W*_*ii*_ had little variance around its expected value of −0.5 [in $N\left(-0.5,\frac{1}{d_{ii}^{\prime 2}}\right)$]. This implied that $exp\left(d_{ii}^{\prime 2}W_{ii}\right)>0$ was a small quantity, which resulted in *p*_*i*_(*T*) being negligible. A similar intuition was applied to display locations close to *i*, because they still had a high degree of visibility. Future experiments with longer scanpaths and more room for potential re-fixations must serve to challenge these predictions. Crucially, Bayesian frameworks were highly interpretable and connected our work to other efforts in modeling top-down influences in perception and decision-making.

It is also important to note that, although the (Najemnik and Geisler, 2005) model was an insightful and influential proposal, to our knowledge, our work is the first to implement it to predict eye movements during visual search in natural images. It is a leap in terms of applications because prior work on Bayesian models was done in very constrained artificial environments (e.g., looking for a tiny Gabor patch embedded in background 1/*f* noise; Najemnik and Geisler, 2005). Moreover, we addressed possible modifications when considering the complexities of natural images, such as the addition of a saliency map as prior, the introduction of two plausible modifications of the template response's mean, and shift in visibility. We also simplified assumptions from Najemnik and Geisler (2005) by not having to measure each person's visibility map beforehand. We used the same visibility map across subjects, which also avoided a potential leak of information about the viewing patterns to the model. Finally, we also shared both an optimized code for the models, which would be useful for others who wanted to replicate the results shown here, and the human dataset of visual search.

Our model aligns with the work of many researchers who proposed probabilistic solutions to model human behavior from first principles. For instance, the authors of Bruce and Tsotsos (2006) and Bruce and Tsotsos (2009), proposed a saliency model based on the information maximization principle, which demonstrated great efficacy in predicting fixation patterns across both pictures and movies. Rasouli and Tsotsos (2014) proposed a probabilistic model that extracted visual features in the form of a saliency map and target characteristics to predict potential target locations in an autonomous 3D vehicle. They showed that the incorporation of attention mechanisms (like saliency maps) improved vehicle performance by reducing the time it took to find the target. Also, Ma et al. (2011) implemented a near-optimal visual search model for a fixed-gaze search task (i.e., exploring the allocation of covert attention). This extended previous models to deal with the reliability of visual information across items and displays, and this model proposed an implementation of how information should be combined across objects and spatial location through marginalization. Interestingly, in both attempts to explain overt and covert allocation of attention, they proposed an implementation through physiologically plausible neural networks.

More generally, the present work expands the general growing notion of the brain as an organ capable of generalizing and performing inferences in noisy and cluttered scenarios through Bayesian inference by building complete and abstract models of its environment. Currently, those models cover a broad spectrum of perceptual and cognitive functions, such as decision making and confidence, learning, multisensorial perception, and others (Knill and Pouget, 2004; Tenenbaum et al., 2006; Meyniel et al., 2015).

## Data Availability Statement

The original contributions presented in the study are included in the article/Supplementary Material and in https://github.com/gastonbujia/VisualSearch, further inquiries can be directed to the corresponding author/s.

## Ethics Statement

The studies involving human participants were reviewed and approved by Comite de Etica del Centro de Educacion Medica e Investigaciones Clinicas Norberto Quirno (CEMIC) [qualified by the Department of Health and Human Services (HHS, USA): IRb00001745—IORG 0001315; Protocol number 435]. The patients/participants provided their written informed consent to participate in this study.

## Author Contributions

MS, GS, and JK designed the task, collected the human data, and defined the model idea. MS prepared the indoor images/targets dataset and coded the first implementation of the presented models, including numerical optimizations and speed-ups. GB and SV pruned the model's code, extended it, and explored their parameters. MS, GB, and JK performed the analysis and wrote the manuscript. All authors reviewed the manuscript and contributed to the article and approved the submitted version.

## Funding

The authors were supported by the CONICET and the University of Buenos Aires (UBA). The research was supported by the CONICET (PIP 11220150100787CO), the ARL (Award W911NF1920240), and the National Agency of Promotion of Science and Technology (PICT 2018-2699).

## Conflict of Interest

The authors declare that the research was conducted in the absence of any commercial or financial relationships that could be construed as a potential conflict of interest.

## Publisher's Note

All claims expressed in this article are solely those of the authors and do not necessarily represent those of their affiliated organizations, or those of the publisher, the editors and the reviewers. Any product that may be evaluated in this article, or claim that may be made by its manufacturer, is not guaranteed or endorsed by the publisher.

## References

[B1] Boccignone G. Cuculo V. D'Amelio A. (2019). How to look next? A data-driven approach for scanpath prediction, in Formal Methods. FM 2019 International Workshops. FM 2019. Lecture Notes in Computer Science, Vol. 12232 (Cham: Springer), 131–145. 10.1007/978-3-030-54994-7_10

[B2] Borji A. Itti L. (2014). Defending Yarbus: eye movements reveal observers' task. J. Vis. 14:29. 10.1167/14.3.29 24665092

[B3] Borji A. Tavakoli H. R. Sihite D. N. Itti L. (2013). Analysis of scores, datasets, and models in visual saliency prediction, in Proceedings of the IEEE International Conference on Computer Vision (Sydney), 921–928. 10.1109/ICCV.2013.118

[B4] Bradley C. Abrams J. Geisler W. (2014). Retina-v1 model of detectability across the visual field. J. Vis. 14:22. 10.1167/14.12.22 25336179 PMC4204678

[B5] Brainard D. H. (1997). The psychophysics toolbox. Spat. Vis. 10, 433–436. 10.1163/156856897X00357 9176952

[B6] Bruce N. Tsotsos J. (2006). Saliency based on information maximization, in Advances in Neural Information Processing Systems (Vancouver), 155–162.

[B7] Bruce N. D. Tsotsos J. K. (2009). Saliency, attention, and visual search: an information theoretic approach. J. Vis. 9:5. 10.1167/9.3.5 19757944

[B8] Bylinskii Z. Judd T. Oliva A. Torralba A. Durand F. (2018). What do different evaluation metrics tell us about saliency models? IEEE Trans. Pattern Anal. Mach. Intell. 41, 740–757. 10.1109/TPAMI.2018.2815601 29993800

[B9] Castelhano M. S. Henderson J. M. (2007). Initial scene representations facilitate eye movement guidance in visual search. J. Exp. Psychol. 33:753. 10.1037/0096-1523.33.4.753 17683226

[B10] Cerf M. Harel J. Einhäuser W. Koch C. (2008). Predicting human gaze using low-level saliency combined with face detection, in Advances in Neural Information Processing Systems (Vancouver), 241–248.

[B11] Cornia M. Baraldi L. Serra G. Cucchiara R. (2016). A deep multi-level network for saliency prediction, in 2016 23rd International Conference on Pattern Recognition (ICPR) (Cancun), 3488–3493. 10.1109/ICPR.2016.7900174

[B12] Cornia M. Baraldi L. Serra G. Cucchiara R. (2018). Predicting human eye fixations *via* an LSTM-based saliency attentive model. IEEE Trans. Image Process. 27, 5142–5154. 10.1109/TIP.2018.2851672 29994710

[B13] Dewhurst R. Nyström M. Jarodzka H. Foulsham T. Johansson R. Holmqvist K. (2012). It depends on how you look at it: Scanpath comparison in multiple dimensions with multimatch, a vector-based approach. Behav. Res. Methods 44, 1079–1100. 10.3758/s13428-012-0212-2 22648695

[B14] Eckstein M. P. Drescher B. A. Shimozaki S. S. (2006). Attentional cues in real scenes, saccadic targeting, and Bayesian priors. Psychol. Sci. 17, 973–980. 10.1111/j.1467-9280.2006.01815.x 17176430

[B15] Gottlieb J. Oudeyer P.-Y. (2018). Towards a neuroscience of active sampling and curiosity. Nat. Rev. Neurosci. 19, 758–770. 10.1038/s41583-018-0078-0 30397322

[B16] Gupta S. K. Zhang M. Wu C.-C. Wolfe J. M. Kreiman G. (2021). Visual search asymmetry: deep nets and humans share similar inherent biases. Advances in Neural Information Processing Systems (Vancouver, WA), 34, 6946–6959. Available online at: https://proceedings.neurips.cc/paper/2021/file/37f0e884fbad9667e38940169d0a3c95-Paper.pdf PMC9436507 36062138

[B17] He K. Zhang X. Ren S. Sun J. (2016). Deep residual learning for image recognition, in Proceedings of the IEEE Conference on Computer Vision and Pattern Recognition (Las Vegas, NV), 770–778. 10.1109/CVPR.2016.90

[B18] Hoppe D. Rothkopf C. A. (2019). Multi-step planning of eye movements in visual search. Sci. Rep. 9, 1–12. 10.1038/s41598-018-37536-0 30644423 PMC6333838

[B19] Itti L. Koch C. (2000). A saliency-based search mechanism for overt and covert shifts of visual attention. Vis. Res. 40, 1489–1506. 10.1016/S0042-6989(99)00163-7 10788654

[B20] Itti L. Koch C. (2001). Computational modelling of visual attention. Nat. Rev. Neurosci. 2, 194–203. 10.1038/35058500 11256080

[B21] Itti L. Koch C. Niebur E. (1998). A model of saliency-based visual attention for rapid scene analysis. IEEE Trans. Pattern Anal. Mach. Intell. 20, 1254–1259. 10.1109/34.730558

[B22] Jarodzka H. Holmqvist K. Nyström M. (2010). A vector-based, multidimensional scanpath similarity measure, in Proceedings of the 2010 Symposium on Eye-Tracking Research & Applications (Austin, TX), 211–218. 10.1145/1743666.1743718

[B23] Judd T. Durand F. Torralba A. (2012). A Benchmark of Computational Models of Saliency to Predict Human Fixations. MIT Technical Report. Available online at: http://saliency.mit.edu/

[B24] Kleiner M. Brainard D. Pelli D. Ingling A. Murray R. Broussard C. (2007). What's new in psychtoolbox-3? Perception. 36:1.

[B25] Knill D. C. Pouget A. (2004). The Bayesian brain: the role of uncertainty in neural coding and computation. Trends Neurosci. 27, 712–719. 10.1016/j.tins.2004.10.007 15541511

[B26] Kotowicz A. Rutishauser U. Koch C. (2010). Time course of target recognition in visual search. Front. Hum. Neurosci. 4:31. 10.3389/fnhum.2010.00031 20428512 PMC2859879

[B27] Kummerer M. Wallis T. S. Bethge M. (2018). Saliency benchmarking made easy: separating models, maps and metrics, in Proceedings of the European Conference on Computer Vision (ECCV) (Munich), 770–787. 10.1007/978-3-030-01270-0_47

[B28] Kummerer M. Wallis T. S. Gatys L. A. Bethge M. (2017). Understanding low-and high-level contributions to fixation prediction, in Proceedings of the IEEE International Conference on Computer Vision (Venice), 4789–4798. 10.1109/ICCV.2017.513

[B29] Ma W. J. Navalpakkam V. Beck J. M. Van Den Berg R. Pouget A. (2011). Behavior and neural basis of near-optimal visual search. Nat. Neurosci. 14:783. 10.1038/nn.2814 21552276 PMC3713779

[B30] Meyniel F. Sigman M. Mainen Z. (2015). Confidence as Bayesian probability: from neural origins to behavior. Neuron 88, 78–92. 10.1016/j.neuron.2015.09.039 26447574

[B31] Najemnik J. Geisler W. S. (2005). Optimal eye movement strategies in visual search. Nature 434, 387–391. 10.1038/nature03390 15772663

[B32] Oliva A. Torralba A. (2006). Building the gist of a scene: the role of global image features in recognition. Prog. Brain Res. 155, 23–36. 10.1016/S0079-6123(06)55002-2 17027377

[B33] O'Reilly J. X. Jbabdi S. Behrens T. E. (2012). How can a Bayesian approach inform neuroscience? Eur. J. Neurosci. 35, 1169–1179. 10.1111/j.1460-9568.2012.08010.x 22487045

[B34] Rasouli A. Tsotsos J. K. (2014). Visual saliency improves autonomous visual search, in 2014 Canadian Conference on Computer and Robot Vision (Montreal), 111–118. 10.1109/CRV.2014.23

[B35] Riche N. Duvinage M. Mancas M. Gosselin B. Dutoit T. (2013). Saliency and human fixations: state-of-the-art and study of comparison metrics, in Proceedings of the IEEE International Conference on Computer Vision (Sydney), 1153–1160. 10.1109/ICCV.2013.147

[B36] Rohe T. Noppeney U. (2015). Cortical hierarchies perform Bayesian causal inference in multisensory perception. PLoS Biol. 13:e1002073. 10.1371/journal.pbio.1002073 25710328 PMC4339735

[B37] Rolfs M. (2015). Attention in active vision: a perspective on perceptual continuity across saccades. Perception 44, 900–919. 10.1177/0301006615594965 26562908

[B38] Russell B. C. Torralba A. Murphy K. P. Freeman W. T. (2008). Labelme: a database and web-based tool for image annotation. Int. J. Comput. Vis. 77, 157–173. 10.1007/s11263-007-0090-8

[B39] Samad M. Chung A. J. Shams L. (2015). Perception of body ownership is driven by Bayesian sensory inference. PLoS ONE 10:e117178. 10.1371/journal.pone.0117178 25658822 PMC4320053

[B40] Schwartenbeck P. Passecker J. Hauser T. U. FitzGerald T. H. Kronbichler M. Friston K. J. (2019). Computational mechanisms of curiosity and goal-directed exploration. eLife 8:e41703. 10.7554/eLife.41703 31074743 PMC6510535

[B41] Simonyan K. Zisserman A. (2014). Very deep convolutional networks for large-scale image recognition. arXiv:1409.155. 10.48550/arXiv.1409.1556

[B42] Tatler B. W. Baddeley R. J. Gilchrist I. D. (2005). Visual correlates of fixation selection: effects of scale and time. Vis. Res. 45, 643–659. 10.1016/j.visres.2004.09.017 15621181

[B43] Tatler B. W. Wade N. J. Kwan H. Findlay J. M. Velichkovsky B. M. (2010). Yarbus, eye movements, and vision. i-Perception 1, 7–27. 10.1068/i0382 23396904 PMC3563050

[B44] Tenenbaum J. Griffiths T. Kemp C. (2006). Theory-based Bayesian models of inductive learning and reasoning. Trends Cogn. Sci. 10, 309–318. 10.1016/j.tics.2006.05.009 16797219

[B45] Torralba A. Oliva A. Castelhano M. S. Henderson J. M. (2006). Contextual guidance of eye movements and attention in real-world scenes: the role of global features in object search. Psychol. Rev. 113:766. 10.1037/0033-295X.113.4.766 17014302

[B46] Torralba A. Sinha P. (2001). Statistical context priming for object detection, in Proceedings Eighth IEEE International Conference on Computer Vision. ICCV 2001, Vol. 1 (Vancouver), 763–770. 10.1109/ICCV.2001.937604

[B47] Turgeon M. Lustig C. Meck W. H. (2016). Cognitive aging and time perception: roles of Bayesian optimization and degeneracy. Front. Aging Neurosci. 8:102. 10.3389/fnagi.2016.00102 27242513 PMC4870863

[B48] Wang Z. Bovik A. C. Sheikh H. R. Simoncelli E. P. (2004). Image quality assessment: from error visibility to structural similarity. IEEE Trans. Image Process. 13, 600–612. 10.1109/TIP.2003.819861 15376593

[B49] Wiecki T. V. Poland J. Frank M. J. (2015). Model-based cognitive neuroscience approaches to computational psychiatry: clustering and classification. Clin. Psychol. Sci. 3, 378–399. 10.1177/2167702614565359

[B50] Wolfe J. M. Horowitz T. S. (2017). Five factors that guide attention in visual search. Nat. Hum. Behav. 1, 1–8. 10.1038/s41562-017-0058 PMC9879335 36711068

[B51] Yang S. C.-H. Lengyel M. Wolpert D. M. (2016a). Active sensing in the categorization of visual patterns. Elife 5:e12215. 10.7554/eLife.12215 26880546 PMC4764587

[B52] Yang S. C.-H. Wolpert D. M. Lengyel M. (2016b). Theoretical perspectives on active sensing. Curr. Opin. Behav. Sci. 11, 100–108. 10.1016/j.cobeha.2016.06.009 30175197 PMC6116896

[B53] Yang Z. Huang L. Chen Y. Wei Z. Ahn S. Zelinsky G.. (2020). Predicting goal-directed human attention using inverse reinforcement learning, in Proceedings of the IEEE/CVF Conference on Computer Vision and Pattern Recognition (Virtual), 193–202. 10.1109/CVPR42600.2020.00027 PMC8218821 34163124

[B54] Yarbus A. L. (ed). (1967). Eye movements during perception of complex objects, in Eye Movements and Vision (New York, NY: Plenum Press), 171–211. 10.1007/978-1-4899-5379-7_8

[B55] Zhang M. Feng J. Ma K. T. Lim J. H. Zhao Q. Kreiman G. (2018). Finding any Waldo with zero-shot invariant and efficient visual search. Nat. Commun. 9, 1–15. 10.1038/s41467-018-06217-x 30213937 PMC6137219

[B56] Zhou Y. Yu Y. (2021). Human visual search follows a suboptimal Bayesian strategy revealed by a spatiotemporal computational model and experiment. Commun. Biol. 4, 1–16. 10.1038/s42003-020-01485-0 33397998 PMC7782508

