# Supplementary material for: Modeling Human Visual Search in Natural Scenes: A Combined Bayesian Searcher and Saliency Map Approach
Source: Front Syst Neurosci. 2022 May 27;16:882315. doi: 10.3389/fnsys.2022.882315 (PMC9197262; doi:10.3389/fnsys.2022.882315)
Supplement: Supplementary file 1 [file Data_Sheet_1.pdf]

Supplementary Material

1 BAYESIAN MODELS

1.1 Next fixation decision

Each of the three main models considered in this work -*sIBS*, *cIBS* and *IBS*- use the same framework described in the model section of the paper. As in the paper, we will follow the original notation introduced in Najemnik and Geisler (2005).

In the eq. 3 we originally described how the Ideal Bayesian Searcher calculates the posteriors on each possible location i for the T-th fixation:

$$p_i(T) = \frac{prior(i) \cdot \prod_{t=1}^{T} exp\left(d'^2_{ik(t)} W_{ik(t)}\right)}{\sum_{j=1}^{n} prior(j) \cdot \prod_{t=1}^{T} exp\left(d'^2_{jk(t)} W_{jk(t)}\right)} \tag{S1}$$

The searcher chooses as next fixation the location that, given the current knowledge of the posteriors (eq. S1), maximizes the probability of identifying the target correctly after the fixation (eq. 1). To find this position, we conditioned on the unknown target location i (eq. 2) resulting in:

$$k_{opt}(T+1) = \underset{k(T+1)}{\arg\max} \left\{ \sum_{i=1}^{n} p_i(T) p(C|i, k(T+1)) \right\} \tag{S2}$$

In order to calculate $k_{opt}(T+1)$, we need to estimate $p(C|i, k(T+1))$ for each possible target location i. This is the probability of identifying correctly the target at location i given the observer being on location $k(T+1)$ after making the next fixation. The decision rule that would maximize the accuracy would be picking the location that has maximum posterior probability on that fixation $k(T+1)$. Using this decision rule, we could write this probability as :

$$p(C|i, k(T+1)) = p(p_i(T+1) \geq p_1(T+1), \ldots, p_i(T+1) \geq p_n(T+1)|i, k(T+1)) \tag{S3}$$

From equation S1, S3 and, the definition of the template response $W_{ik(t)}$ in eq. 4, Najemnik and Geisler derive the following expression for $p(C|i, k(T+1))$:

$$p(C|i, k(T+1)) = \int_{-\infty}^{\infty} \phi(w) \prod_{j \neq i} \Phi\left(\frac{-2\ln(\frac{p_j(T)}{p_i(T)}) + d'^2_{jk(T+1)} + 2d'_{ik(T+1)}w + d'^2_{ik(T+1)}}{2d'_{jk(T+1)}}\right) dw \tag{S4}$$

where ϕ represents the density function of a standard normal distribution and Φ is the left cumulative distribution function. Finally, for each potential next fixation location $k(T+1)$ we can calculate

$\sum_{i=1}^{n} p_i(T)p(C|i, k(T+1))$ and build a *detection map* (second row maps in S1) where the model will select the fixation according to equation S2.

It is important to mention that the original deduction of equation S4 was made considering the original template response definition proposed for the IBS model. We did not deduce this equation for each new definition of the template response. Instead, we use it with the definition of W that responds to each different model as stated in eq. 5, 6 and 7.

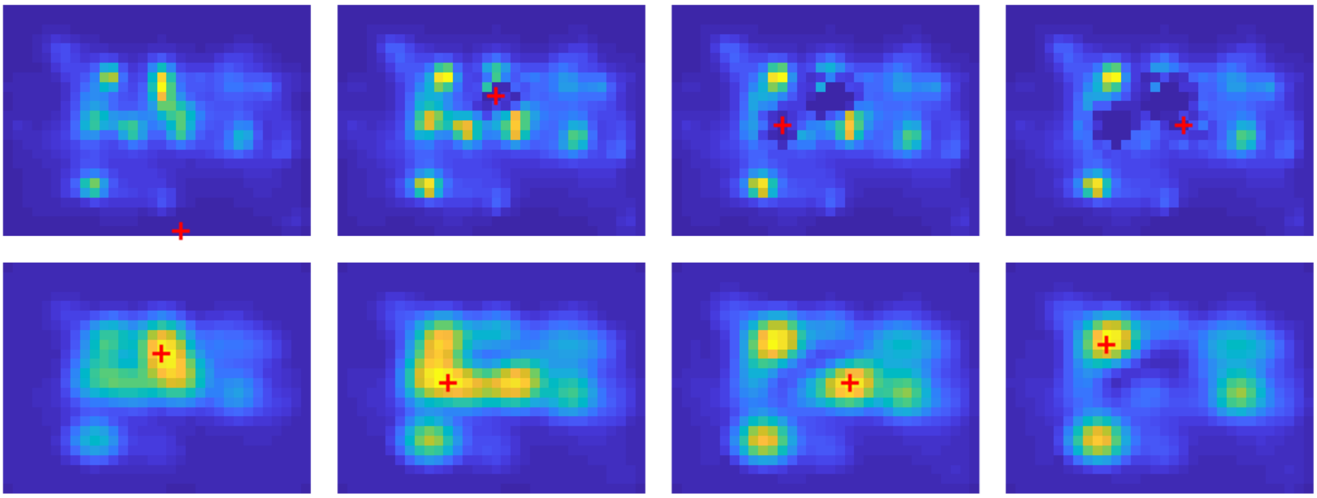

Figure S1. Model search example Example of the first four saccades of sIBS model for the image `grayscale_11_oliva.jpg`. Each column is the calculation of a saccade, with the initial fixation previous to the saccade on the first row represented as a red cross, and the final position on the second row. On the first row, the heat map is the probability of finding the target on that fixation (eq. S1). On the second row, the heat map is the detection map for the next saccade and the maximum (the next fixation) is marked with a red cross.

1.2 Implementation details

The model was implemented in MATLAB R2018b and it was tested on an Intel i7 8700 @ 3.20GHz. Here we show a pseudo-code of the main algorithm 1 that performs the visual search for the three main models, sIBS, cIBS and IBS. The main algorithm also reduces the original image to a grid of size $\delta = 32$px. subtending an image size of 32×24. To simplify notation, each region is numbered from $i = 1$ to 32×24.

The visibility map $d' \sim \mathcal{N}(\mu, \mathbf{\Sigma})$ was centered on each fixation point (μ is the 2D-coordinate in pixels), and its covariance was $\mathbf{\Sigma} = \begin{pmatrix} 2600 & 0 \\ 0 & 4000 \end{pmatrix}$pxs^2.

For more details, the code can be found in https://github.com/gastonbujia/VisualSearch.

Algorithm 1: Bayesian model

Input: *Img*, *Target*, (x, y), T_{max}, *random state = 1234*
Output: scanpath *k*

prior $\leftarrow$ saliency(*Img*)
$d' \leftarrow$ visibilityMap $\sim \mathcal{N}(\mu, \Sigma)$
$W \leftarrow templateResponse($*Img*, *Target*$)$
$k(1) \leftarrow (x, y)$

main cycle
for $T \leftarrow 1$ **to** T_{max} **do**
  compute posteriors for each location i
  for $i \leftarrow 1$ **to** n **do**
    $p_i(T) \leftarrow \frac{prior(i) \cdot \prod_{t=1}^{T} exp\left(d'^2_{ik(t)} W_{ik(t)}\right)}{\sum_{j=1}^{n} prior(j) \cdot \prod_{t=1}^{T} exp\left(d'^2_{jk(t)} W_{jk(t)}\right)}$
  end
  compute next fixation
  $k(T+1) \leftarrow nextFix(k, T, p, W, d')$
  if *target found* **then**
    exit **for** loop
  end
end

The algorithm 1 has several subrutines beeing the most relevants: 1) `nextFix` and 2) `templateResponse`. The first one has the bigger computational complexity (at least $\mathcal{O}(n^2)$ where n is the amount of possible locations) which implements the calculations corresponding to eq. S4 needed to find the next fixation location by computing the detectability map.

`nextFix`(*args*)
Data: k, T, p, W, d'
Result: next fixation location $k(T+1)$
for $k_{T+1} \leftarrow 1$ **to** n **do**
  for $i \leftarrow 1$ **to** n **do**
    $$p(C|i, k_{T+1}) \leftarrow \int_{-\infty}^{\infty} \phi(w) \prod_{j \neq i} \Phi\left(\frac{-2\ln(\frac{p_j(T)}{p_i(T)}) + d'^2_{jk_{T+1}} + 2d'_{ik_{T+1}} w + d'^2_{ik_{T+1}}}{2d'_{jk_{T+1}}}\right) dw$$
  end
  detectabilityMap(k_{T+1}) $\leftarrow \sum_{i=1}^{n} p_i(T) p(C|i, k_{T+1})$
end
$k(T+1) \leftarrow \arg\max$ detectabilityMap

The `templateResponse` computes W depending on the model. W is represented as tensor $W(i, j)$ that represents the evidence gathered for each possible location i given that the subject is fixating on location j (eq. 6). We pre-computed W for all possible fixations locations. This function is also responsible for computing the similarity map ($\phi(i)$ in eq. 6).

`templateResponse(`*args*`)`
Data: $Image, Target, Model, d'$
Result: W
for $i \leftarrow 1$ **to** n **do**
  for $j \leftarrow 1$ **to** n **do**
    $\tilde{\mu}_{ij} \leftarrow \mathbb{1}_{(i=\text{target location})} - 0.5$
    if *sIBS* **then**
      $\tilde{\mu}_{ij} \leftarrow \tilde{\mu}_{ij} \cdot (d'_{ij} + \frac{1}{2}) + \text{SSIM}(i) \cdot (\frac{3}{2} - d'_{ij})$
    end
    if *cIBS* **then**
      $\tilde{\mu}_{ij} \leftarrow \tilde{\mu}_{ij} \cdot (d'_{ij} + \frac{1}{2}) + \text{CrossCorr}(i) \cdot (\frac{3}{2} - d'_{ij})$
    end
    $\tilde{\sigma}_{ij} \leftarrow \frac{1}{a \cdot d'_{ij} + b}$
    draw $W(i, j)$ from $\mathbf{W} \sim \mathcal{N}(\tilde{\mu}_{ij}, \tilde{\sigma}^2_{ij})$
  end
end

2 TARGET DISTRIBUTION

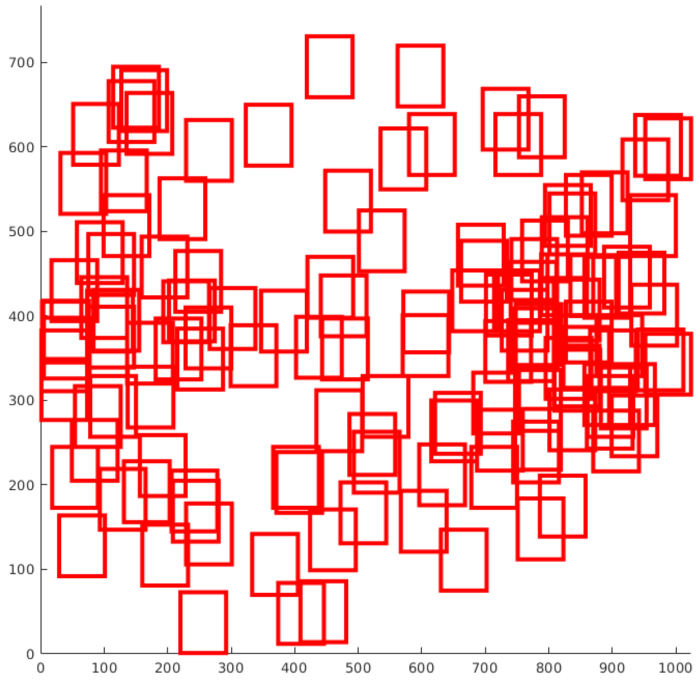

Figure S2. Targets distribution

REFERENCES

Najemnik, J. and Geisler, W. S. (2005). Optimal eye movement strategies in visual search. *Nature* 434, 387–391
